# Supplementary material for: Disturbed neurovascular coupling in hemodialysis patients
Source: PeerJ. 2020 Apr 15;8:e8989. doi: 10.7717/peerj.8989 (PMC7166048; doi:10.7717/peerj.8989)
Supplement: Supplemental Information 1 — Data imagings (T map), subjects and clinical variables (CSV format) and software for nii format (mricron). [file peerj-08-8989-s001.zip › raw_data/software_for_nii_format/mricron/html/bat.html]

MRIcron Batch File Page


|  |  |
| --- | --- |
|  | MRIcron Advanced Settings |

**Advanced Options**

**Command line options**   Usage: mricron BackgroundImageFilename [options]

If you launch MRIcron from the command line you can (optionally)
include default parameters to specify desired settings. Nice examples
of these commands are the batch files that are created in the same
folder as the mricron.exe program (you need to include the 'Tutorials'
when you install mricron). For example, lets look at simple batch file:  
  start /MAX mricron .\templates\ch2bet.nii.gz -c -0 -l 20 -h 140  
The terms 'start /MAX mricron' are standard parts of a Windows batch
file. This launches MRIcron and ensures that the main window is
maximized to fill the entire screen. You could run this from a batch
script (a text file with the name '.bat') or from the command line
(e.g. choose Start/Run and type 'cmd' to start the Windows command
line). Note that this script will only work from the folder where
MRIcron is stored. Otherwise, the script will need to specify the
location of the software:  
  start /MAX c:\mricron\mricron c:\mricron\templates\ch2bet.nii.gz -c -0 -l 20 -h 140 x  
Note that after the program name the script specifies the background
image that is to be loaded, in this cas the image ch2bet.nii.gz. After
this, you can optinally specify additional settings. For example, the
"-c -0" sets the image to have a grayscale color scheme. While "-l 20
-h 140" sets the image brightness to be set for the range 20..140 (e.g.
voxels with values less than 20 will appear black, voxels greater than
140 will be white, and intermediate values will be linearly scaled for
this range). Finally, the 'X' command adjusts the adjusts the
proportions of the sagittal, coronal and axial panels so that each of
these views will be shown at a similar scale.   
This next example shows how you can load an overlay on top of another image:  
  start mricron .\templates\ch2.nii.gz -c -0 -l 20 -h 140 -o .\templates\ch2bet.nii.gz -c -1 10 -h 130  
Note how this script uses some parameters multiple times - the first
set of -c-l-h parameters refer to the background image (ch2), while the
second set refer to the overlay image (ch2bet). The parameters that can
be adjusted for each image are noted with an asterix in the table below
(-c, -l, -h, -z).

Here is a complete list of the parameters you can specify

|  |  |  |
| --- | --- | --- |
| **Parameter** | **Notes** |  |
| -b <%> | Sets transparency of overlays on background. Supported values are -1,0,20,40,50,60,80,100. A value of -1 signifies additive color blending. |  |
| -c <LUT name> | Specify color lookup table. "-c bone" will load bone.lut. You can also specify the index number of the LUT by using the prefix "-". For example, "-c -1" will load the red color scheme, while "-c -0" will load the grayscale color scheme. | \* |
| -d | Defaults will be saved when the user quits. |  |
| -f | Loads image 'flat': the NIfTI orientation matrix is ignored. This is the same effect as editing the .ini file and setting 'Reslice=0' |  |
| -h | Sets maximum value for image intensity scaling. For example a Z-score statistical map loaded with "-l 1.96 -h 4" will show values near 1.96 as maximal dark and values near 4 as maximal bright. See also -l and -z | \* |
| -l | Sets minimum value for image intensity scaling. see -h | \* |
| -m | Will show a multi-slice view using default multislice settings. |  |
| -m <INI name> | Will show a multi-slice view using the settings for the file <INI name>. For example "-m c:\mymulti.ini". Multislice settings files can be created by selecting File/SaveSettings in the multislice window. |  |
| -o <overlay name> | Will load overlay <overlay name>. For example "-o c:\statmap.hdr". |  |
| -r | Will show a rendering using default rendering settings. |  |
| -r <INI name> | Will show a rendering using the settings for the file <INI name>. For example "-r c:\myrender.ini". Rendering settings files can be created by selecting File/SaveSettings in the render window. |  |
| -s <v> | Settings smoothing. <v> should be a value 0..3. If <v> is odd (1 or 3) then bilinear smoothing is applied to images (otherwise, nearest neighbor is used). If <v> is 2 or 3 then overlays will be scaled using trilinear interpolation (otherwise, nearest neighbor is used). |  |
| -t <%> | Sets transparency of overlays with respect to other overlays. Supported values are -1,0,20,40,50,60,80,100. A value of -1 signifies additive color blending. |  |
| -v <drawing name> | Will load volume of interest <drawing name>. For example "-o c:\lesion.voi". |  |
| -x | Main window will be maximized to fill entire screen. The size of each view (sagittal, coronal and axial) will be proportionally scaled. |  |
| -z | Image color scaling will be from zero. For example a Z-score statistical map loaded with "-l 1.96 -h 4 -z" will hide values below 1.96, but will show values near 1.96 as a medium intensity and values near 4 as maximal bright. See also -h | \* |

**Default Settings**

You can adjust many of MRIcron's settings by choosing Help/Preferences. A window will appear that allows you to edit many of the values from your mricron.ini file. The window is shown below, and the mricron.ini settings are described in the table below.

MRIcron remembers users preferences. Three standard .INI format text
files are automatically generated: mricron.ini, \render\default.ini,
and \multislice\default.ini. The file mricron.ini saves general
settings, while the other files store values specific to the rendering
and multislice views respectively. The user can save custom multislice
and rendering settings by opening up the render (or multislice view)
and creating their desired settings and then choosing
File\SaveSettings. Deleting the ini files will return the software to
its factory settings.

The mricron.ini file has a few values that can not be adjusted from
the program, but can be changed by editing the file with a text editor.
Here is a complete list of the parameters you can specify

|  |  |
| --- | --- |
| **Parameter** | **Notes** |
| file0...file5= | Stores the five most recently viewed images. These will appear in the File/OpenRecent submenu. |
| reslice= | If 1, image will be spatially oriented using the NIfTI transforms, similar to SPM5. If 0, images will be shown as saved to disk (like SPM2 and FSLview). Reslice=1 requires more memory, but hopefully avoids left-right confusions. |
| ShowDraw= | If 1, the 'Draw' menu and drawing tools will be visible. |
| Smooth= | If 1, resliced images will use the slow but relatively precise trilinear interpolation |
| XBar= | If 1, cross-hairs will mark the currently selected voxel. |
| OverlaySmooth= | If 1, overlays will be scaled to background image using smooth trilinear interpolation. If 0, overlays will use nearest neighbor (and the edges may appear jagged). |
| LRMirror= | If 1, images with NIfTI orientation parameters will appear in radiological convention (with left on the right). If 0, images will be in neurological convention (with left on the left). |
| Yoke= | If 1, images in multiple instances of MRIcron will show the same location - changing the selected voxel in one instance will cahange the view in other instances. |
| MaxDim= | If an image is larger than this value in any dimension, the image will be rescaled so that this is the largest dimension. This minimizes memory consumption. For example, if this is set to 256 and you open an image with 512x512x300 voxels, it will be displayed as 256x256x150voxels. |
| Zoom= | Default image scaling. Zero specifies that the image is strecthed to fit the window, 1 for best integer fit to window (e.g. if a 235% scaling is possible, the image will be scaled 200%), 2 for 100%, 3 for 200%, etc. |
| LUT= | Selected color scheme. E.G. if 24, then the 24th color scheme is used. |
| XBarGap= | Empty gap between crosshairs. If 7, then 7 pixels separate crosshairs. |
| XBarThick= | Width of crosshairs. |
| XBarClr= | Color of crosshairs. This is a 24-bit value, with top 8 bytes representing blue, middle 8 representing blue and least significant 8 representing red. So a value of 255 would be a bright, pure red. |
| VOIClr= | Color of volume of interest drawing. Same values as XBarClr. |
| BGTransPct= | Transparency of overlays on background (in percent). 0 for opaque overlays, 50 for a translucent overlay (50% each) and 100 for a completely transparent overlay. -1 specificies additive combination. |
| OverlayTransPct= | Transparency of overlays relative to each other. Same values as BGTransPct |
| MaxThreads= | Maximum number of CPU cores used for rendering. If set to 5, only five cores will be used regardless of the number of CPUs. Higher numbers lead to faster rendering, but can slow other applications. For systems with more than two cores, a good value is the number of cores minus one. For example, with a quad-CPU machine, set this to 3. If you have fewer CPUs than MaxThreads, MRIcron will use all of your available CPUs (e.g. threads will equal the number of CPUs), offering optimal speed. |
| SigDigits= | Number of digits shown after decimal place. Influences the voxel intensity value shown in the bottom-left status label. |
| TabletPressure= | Only used when using a Tablet device (e.g. Wacom tablet or a Tablet PC which pressure sensitive stylus). Threshold for transitioning between a thin (1 voxel) and thick (3 voxel) pen - if pen pressure is double this threshold, a five pixel line will be drawn.. Tablet stylus pressure will range from 0..100. If set to 100, the pen will always draw a thin line, regardless of pressure. If set to 30, light pressure (<30%) will create a thin line, medium pressure (30..60%) will lead to a 3-voxel line and heavy pressure (>60%) will causes a very thick line (5 pixels wide). |
| TabletErasePressure= | Pressure threshold for stylus eraser on Tablet systems. Same comments as TabletPressure. |
| LesionSmooth= | Smoothing Full Width Half Maximum (in mm) This is used by the Draw Menu's "Create SPM5 mask" function. |

  

|  |
| --- |
|  |
