# Supplementary material for: Disturbed neurovascular coupling in hemodialysis patients
Source: PeerJ. 2020 Apr 15;8:e8989. doi: 10.7717/peerj.8989 (PMC7166048; doi:10.7717/peerj.8989)
Supplement: Supplemental Information 1 — Data imagings (T map), subjects and clinical variables (CSV format) and software for nii format (mricron). [file peerj-08-8989-s001.zip › raw_data/software_for_nii_format/mricron/html/dcm2nii.html]

dcm2nii DICOM to NIfTI image conversion


|  |  |  |
| --- | --- | --- |
| |  |  | | --- | --- | |  | dcm2nii DICOM to NIfTI conversion | |

|  |  |  |  |  |  |  |  |  |  |  |  |  |  |  |  |  |  |  |  |  |  |  |  |  |  |  |  |  |  |  |  |  |  |  |  |  |  |  |  |  |  |  |  |  |  |  |  |  |  |  |  |  |  |  |  |  |  |  |  |  |  |  |  |  |  |  |  |  |  |  |  |  |  |  |  |  |  |  |  |  |  |  |  |  |  |
| --- | --- | --- | --- | --- | --- | --- | --- | --- | --- | --- | --- | --- | --- | --- | --- | --- | --- | --- | --- | --- | --- | --- | --- | --- | --- | --- | --- | --- | --- | --- | --- | --- | --- | --- | --- | --- | --- | --- | --- | --- | --- | --- | --- | --- | --- | --- | --- | --- | --- | --- | --- | --- | --- | --- | --- | --- | --- | --- | --- | --- | --- | --- | --- | --- | --- | --- | --- | --- | --- | --- | --- | --- | --- | --- | --- | --- | --- | --- | --- | --- | --- | --- | --- | --- | --- |
|  | **Introduction** Important note: dcm2nii is still beta software - please carefully monitor the output from this software. In particular, be aware of potential left-right flipping. This software is provided under the  BSD license.  DCM2NII attempts to convert images from the proprietary scanner format to the NIfTI format used by FSL, SPM5, MRIcron and many other brain imaging tools. NIfTI is a modern incarnation of the Analyze format, but includes important information like the orientation of the image. DCM2NII is a stand-alone program that is distributed with MRIcron. It is natively compiled for Windows, Linux x86, Mac OSX PPC and Mac OSX x86.   |  |  |  | | --- | --- | --- | | |  |  | | --- | --- | | The NIfTI image format standard was designed for scientific analysis of brain images. The format is simple, compact and versatile. The images can be stored as a pair of files (hdr/img, compliant with most Analyze format viewers), or a single file (nii). Programs like FSL and MRIcron can also read compressed (nii.gz) images. One nice feature about NIfTI is that the format attempts to keep spatial orientation information. Therefore, NIfTI software that can read the spatial information (MRIcron and SPM5) should reduce your chance of making left-right errors. Also, software like SPM5 will tend to be more accurate at coregistering images, as all the images from an individual can use the scanner position as a beginning estimate for alignment. For example, here are two scans from the same individual (the sagittal T1 is shown in grayscale, and the coronal FLAIR is shown in reds). Note that the scans were acquired with different orientations (with the FLAIR along the axis of the hippocampus), however MRIcron shows the image orientation correctly. |  | |   **Installation**   1. Follow the instructions to install MRIcron on your hard disk - this should create a program named dcm2nii.exe (Windows) or dcm2nii (Unix). 2. You can now run the command line program dcm2nii or the graphical program dcm2niigui.   **Hacking the initialization file to fine tune dcm2nii and dcm2niigui**   1. This software will generate a text initialization file when it quits. Therefore, run the program once to enusre that a ini file is created. If you are using Windows, this file is in    the same folder as dcm2nii (e.g. dcm2nii.ini and dcm2niigui.ini). If you are using Unix (Linux, OSX) then    this file is created in a hidden folder in your home directory (e.g. ~/.dcm2niigui/dcm2niigui.ini and ~/.dcm2nii/dcm2nii.ini). 2. Open dcm2nii.ini (or dcm2niigui.ini) with a text editor (double click on the file). You will be able to adjust the settings:      |  |  |    | --- | --- |    | Text | Description |    |    | [BOOL] |  |    | AnonymizeSourceDICOM=0 | If '=1' then dcm2nii will create anonymized copies of DICOM images (remove name, DOB, patient ID, sex). No other conversion will occur. |    | ManualNIfTIConv=1 | If '=1' then dcm2nii will prompt user to describe output subformat for every NIfTI image dragged onto the program. Otherwise, subformat will be based on $D, SingleNIIFile,SPM2 settings. |    | 4D=1 | If '=1' then dcm2nii will generate 4D files (FSL style), otherwise output will be 3D (SPM style). |    | Anonymize=1 | If '=1' then patient name will not be copied to NIfTI header. |    | SingleNIIFile=1 | If '=1' then dcm2nii will create .nii files (FSL style), otherwise .hdr/.img pairs will be created (SPM style) |    | Gzip=0 | If '=1' then dcm2nii will create compressed .nii.gz files (FSL style). |    | SPM2=0 | If '=1' then dcm2nii will create Analyze images (SPM2 style), otherwise headers will be in NIfTI (SPM5/FSL). |    | AppendDate=1 | If '=1' then output filename will include date of study. |    | AppendAcqSeries=1 | If '=1' then output filename will include acquisition number. |    | AppendProtocolName=1 | If '=1' then output filename will include protocol name. |    | AppendPatientName=0 | If '=1' then output filename will include patient name. |    | AppendFilename=0 | If '=1' then output filename will include source filename. |    | EveryFile=1 | If '=1' then all .par/.rec files in the source folder will be converted, otherwise only the file specified will be converted |    | Swizzle4D=1 | If '=1' then DICOM images that are stored in the sequence XYTZ are converted to have the order XYZT (e.g. the first three dimensions are spatial, the fourth is temporal). If set to '=0', then the data is converted as stored in the original DICOM dataset. Note that all NIfTI viewers expect that the first three dimensions are spatial, so it is strongly recommended that you keep the default setting ('=1') |    | Stack3DImagesWithSameAcqNum=0 | By default, dcm2nii creates 4D images from a set of 3D DICOM images that share the same Series number. If this parameter is set to '=1' then sequential 3D Series that share the same Acquisition number will be converted to a single 4D NIfTI volume. In practice, this option often helps convert a set of perfusion images into a 4D volume. |    | RecursiveUseNameAppend=0 | If '=1' then output images will include the name of their folder in their filename. For example, when converting DICOM images in the folder subj1, the resulting NIfTI images will have names like 'subj1s2a3.nii'. This is useful if you want to add anonymized labels to DICOM images. This option only influences DICOM -> NIfTI conversion. |    | CustomRename=0 | If '=1' then you are prompted to manually enter a filename when you convert a NIfTI image to a different version of NIfTI (otherwise, the output image has the prefix 'f' appended to the input filename, to indicate a change in format). Note this option only influences NIfTI->NIfTI conversion, not DICOM->NIfTI conversion. |    | enablereorient=1 | If '=1' then T1 images will be resliced to the nearest orthogonal, so they will be displayed in an intuitive manner when you view these images with software that ignores NIfTI rotation transforms. Note that fMRI (T2\*) and DTI images are not reoriented, as this might interfere with slice-time and gradient direction algorithms, respectively. |    | createoutputfolder=0 | If '=1' then the output NIfTI images will be stored in a folder named after the time of testing and the particiants name. For example, if you scanned John Doe at 8:30am on the 28th of September 2008, the converted images would be stored in a folder named '20080928\_083025JOHNDOE'. |    | CollapseFolders=0 | Normally (when =0), dcm2nii searches each folder independently, for example if you convert c:\folderA and it contains c:\folderA\subfolder1 and c:\folderA\subfolder2, then dcm2nii will convert all the files in subfolder1 and then convert all the files in subfolder2. However, if this value is set to '=1' then all the files in both subfolder1 and subfolder2 will be sorted together and converted in a single step. This latter option is slower and more memory intensive than the previous option. Only use this if your image exporting software distributes scans from the same series into different folders (e.g. if fMRI volumes 1..80 are stored in one folder and 81..160 are stored in a different folder). Note that the depth of searching is controlled by RecursiveFolderDepth (see below). |    | [INT] |  |    | MinReorientMatrix=255 | Images with a larger matrix size than this value will be reoriented to canonical space (see below). |    | MaxReorientMatrix=1023 | Images with a smaller matrix size than this value will be reoriented to canonical space (as long as they are larger than MinReorientMatrix). |    | RecursiveFolderDepth=5 | The software will search for DICOM images in this dept of subfolders. For example, consider the directory 'c:\f0\f1\f2\f3'. If you select f0 as your input folder, and have a search depth of 2, then the software will convert all folders in f0, f1 and f2, but will not look inside f3 (as this would require a search depth of 5). |  3. With each option listed under the heading [BOOL], add    a '1' for yes and a '0' for no. For    example, if you want to have images saved as .hdr/.img pairs, set    SingleNIIFile to 0. If you want to save images a single .nii files, set    this to 1. The append options adjust the output filenames. For example,    if you had both AppendDate and AppendProtocolName set to 1, the    converted images would have names such as 20060331\_123456anat1x1x1.nii    if the data session was acquired on 31 March 2006 at 12:34:56 and the    protocol was called 'anat1x1x1'. The last two values (listed under the    heading [INT]) except integer values - for example if you set    'LastClip=8' then the last 8 volumes of every 4-dimensional dataset    will not be saved (e.g. if you had an fMRI dataset with 120 volumes,    only the first 111 volumes would be converted). A fuller description of    these options is in the gray box below.   **Running DCM2NII**  There are two ways to run dcm2nii   1. You can either drag and drop files onto the program - this will convert the images using the    values in your dcm2nii.ini file (see above). 2. You can    launch dcm2nii from the command line, specifying specifically the    options to use.   To use dcm2nii by dragging and dropping:   1. Place all the DICOM (or Philips PAR/REC) images you wish to convert into a folder where you have write access. 2. Drag and drop one of the images onto dcm2nii. 3. You can now vew the images with MRIcron, SPM5, FSL, or other Analyze/NIfTI viewers.  To see your options for running dcm2nii from the command line, simply execute the program without specifying any files (e.g. just double click on the program to launch it). The available options will the be written to the screen:   |  | | --- | | Chris Rorden's dcm2nii :: 15 Oct 2008  Either drag and drop or specify command line options:     dcm2nii [options] [sourcenames]  OPTIONS:    -a Anonymize [remove identifying information]: Y,N = Y    -b load settings from specified inifile, e.g. '-b C:\set\t1.ini'    -c Collapse input folders: Y,N = N    -d Date in filename [filename.dcm -> 20061230122032.nii]: Y,N = Y    -e events (series/acq) in filename [filename.dcm -> s002a003.nii]: Y,N = Y    -f Source filename [e.g. filename.par -> filename.nii]: Y,N = Y    -g gzip output, filename.nii.gz [ignored if '-n n']: Y,N = Y    -i ID in filename [filename.dcm -> johndoe.nii]: Y,N = Y    -n output .nii file [if no, create .hdr/.img pair]: Y,N = Y    -o Output Directory, e.g. 'C:\TEMP' (if unspecified, source directory is used)    -p Protocol in filename [filename.dcm -> TFE\_T1.nii]: Y,N = Y    -r Reorient image to nearest orthogonal: Y,N    -s SPM2/Analyze not SPM5/NIfTI [ignored if '-n y']: Y,N = N    -v Convert every file in the directory: Y,N = Y   You can also set defaults by editing C:\mricron\dcm2nii.ini    EXAMPLE: dcm2nii -a y -o C:\TEMP C:\DICOM\input1.par C:\input2.par |      **Examples**   - **dcm2niigui -a y -o C:\TEMP C:\DICOM\IM1** Convert all DICOM images in C:\DICOM and place anonymized NIfTI images in the folder C:\TEMP- **dcm2niigui C:\DIR\IM1** Convert all DICOM images in C:\DIR and place NIfTI images in C:\DIR- **dcm2niigui -v n C:\DIR\IM1 C:\DIR\IM2** Convert DICOM files IM1 and IM2 and place NIfTI images in C:\DIR- **dcm2niigui "C:\MY DIR\IM1"** Convert all DICOM images in C:\MY DIR and place NIfTI images in C:\MY DIR. Note that quotation marks are used for file and folder names with spaces (optional for single files, required if you want to specify multiple files).   **Reorienting to canonical space** The NIfTI format stores spatial transforms so that software can determine the orientation of the image. This means that MRIcron can display the image with an intuitive orientation. However, many programs ignore these transforms, and display the images as they are saved to disk (e.g. FSLview, MRIcro) - this means that a sagittally acquired scan appears very differently from an axially acquired scan. In fact, the three spatial dimensions (left-right, anterior-posterior, superior-inferior) can be saved in 48 different orthogonal orientations. The drawing below shows just three of these possible orientations.   You can set dcm2nii to reorient all images so they are all aligned to the nearest orthogonal direction to 'canonical space' (i.e. as close as possible to the rotation matrix [1 0 0; 0 1 0; 0 0 1]). This means programs like FSLview and MRIcro will display the images in a sensible orientation, regardless of the acquisition. The NIfTI transform codes the residual oblique orientations, so no information is lost in this tranformation. However, you will only want to orient anatomical scans to canonical space - reorienting fMRI data can disrupt slice timing correction (as the software assumes that the slice order of the stored data is correlated with the time of acquisition). Reorienting can also disrupt analysis of the DTI data (as the diffusion directions are not adjusted for the change in image orientation). Therefore, the "MinReorientMatrix" allows you to adjust which images will be reoriented - a value of 255 ensures that T1/T2 scans (typically with a 256x256 matrix) are reoriented, while fMRI (~64x64) and DTI (~128x128) scans are not. If you do not want any scans reoriented, reset this to a very large value (e.g. 5000).     After reorienting, dcm2nii will attempt to 'autocrop' T1-weighted anatomical images (images with a Echo Time [TE] of less than 20ms). A new copy of the image is created with the prefix 'c' that attempts to remove excess air surrounding the individual as well as parts of the neck below the cerebellum. This excess neck can disrupt normalization of images (as the template images do not have similar neck regions). This new image has a slightly different NIfTI transform - the origin is adjusted to compensate for the removed portions of the image. The image below shows a T1-weighted scan before and after cropping.     As a final note, reorienting images is useful if you want to create masks for an image to use with SPM or FSL. These programs require that the mask image has precisely the same dimensions as the image it is designed to mask. In these cases, you can not apply the precise spatial transforms to an image (as the oblique orientation corrections means that the resulting drawing will have different dimensions than the original image. Therefore, you will want to draw a mask on on a image that has not used the fine spatial transforms. You can use MRIcro or FSLview to do this (as they ignore these transforms). If yu use MRIcron, select Help/Preferences and uncheck the 'Reorient (reslice) images when loading' option - this will ensure that the raw image is loaded. Regardless of which software you use, having a canonically aligned image will mean that the image will be displayed in a sensible manner.   **NIfTI Sub-Formats** SPM5 and FSL both support NIfTI format images. However, by default these programs assume your data is in slightly different formats. Most SPM5 users generate a single 3D volume for each timepoint, and each image is saved as both a .hdr and .img file (separating the header information from the raw image data). On the other hand, by default FSL uses a single 4D dataset, with all the data stored in a single .nii file (this single file contains both the header and raw image data). To save disk space, FSL saved these files as compressed gzipped files (.nii.gz). Therefore, you may want to convert your DICOM data to a different NIfTI sub-format depending on how you want to process and analyze your data. You should adjust dcm2nii's settings depending on which software you will use for post-processing. Here are some general guidelines:   1. FSL/MRIcron: compressed NIfTI (.nii.gz) - SingleNIIFile=1; Gzip=1; SPM2=0 2. Recent software (SPM5/Voxbo/FSL/MRIcron): NIfTI (.nii) - SingleNIIFile=1; Gzip=0; SPM2=0 3. Legacy software (SPM2/Analyze/MRIcro): analyze (.hdr/.img) - SingleNIIFile=0; Gzip=0; SPM2=1   **Converting between NIfTI Sub-Formats** FSL includes the avwsplit and avwmerge tools for converting between 3D and 4D NIfTI images. This is useful, as FSL likes 4D images while SPM likes 3D images. The latest versions of dcm2nii (since May 2007) can also help you convert between NIfTI subformats. Specifically, if you drag and drop a NIfTI image (.nii, .nii.gz, or .hdr/.img subformats) onto dcm2nii it will ask how you want the data converted. You can convert these files to SPM2 (analyze 3D hdr/img), SPM5 (3D hdr/img), 3D nii, 4D nii, and FSL (NIfTI 4D nii.gz). If ManualNIfTIConv=1 then the user will be prompted for every file to specify the output format, while if this value is ManualNIfTIConv=0 then the files will be converted automatically using the sub-formant specified in the dcm2nii.ini file. Note that this software will both change subformat and/or convert 4D files to 3D files. However, it does not convert 3D files to 4D files (use avwmerge for this).  **Anonymizing DICOM images** This software can also 'anonymize' DICOM data - protecting the participants private information. There are a number of free as well as professional programs that can help anonymize DICOM data. My favorite is the free uniPACS viewer can strip all the private tags from a DICOM file (choose File/BatchFileExport and select 'Anonymize' from the file menu). However, it is worth mentioning that some DICOM images store important data in the 'private' tags - for example, Siemens data includes information about the number of slices in a mosaic as well as DICOM diffusion directions. Therefore, use these 'strong' anonymizers with caution. In contrast, dcm2nii provides a 'weak' anonymization: it only anonymizes the patients name (0010:0010), ID (0010:0020), date of birth (0010:0030), sex (0010:0040), age (0010:1010) and weight (0010:1030). The name is replaced with the number of seconds that elapsed between the study time and January 1, 2000 (ensuring that data from different individuals will not be confused). In theory, the participant can still be identified by study time (if you know when people received scans), and some manufacturers may store personal information in other parts of the DICOM file. To use dcm2nii's DICOM anonymizer, simply edit the dcm2nii file to read "AnonymizeSourceDICOM=1". Then just drag and drop DICOM files on the program - an anonymized file will be created (with the same name as the original image, but with the extension '.dcm' appended at the end). Note that in this mode the software will not convert the DICOM files to the NIfTI format. You may want to keep two copies of dcm2nii with different filenames (and hence different .ini files) - for example you could call one 'dcmanon' and another 'dcm2nii' so that one copy generates anonymized files and the other converts files.   **dcm2niiGUI** I find dcm2nii very easy to use - just drop the images that you wish to convert onto the program's icon. However, some people prefer programs with a graphical user interface. The dcm2niigui program is simply a version of dcm2nii with a graphical interface. Just launch the program, then drag and drop the images you wish to convert. The 'Output format' pulldown menu determines whether the images will be saved in SPM or FSL style NIfTI format. You can also choose help/preferences to more advanced options. In addition, you can use the File/AnonymizeDICOM command to strip personal details from DICOM images. Finally, you can use the File/ModifyNIfTI command to change existing NIfTI images - this command guides you through selecting the images and then choosing how you want to modify the images (remove volumes, changing subformat, reorienting, or changing the order of the 3rd and fourth dimension). For Macintosh OSX users: launch dcm2niigui and then drag the images you wish to convert to the program's icon in the dock, rather than dragging the images to the program itself.    **Performance** Converting DICOM images is fast compared to the other processing stages common to neuroimaging. However, several people have asked me how to improve dcm2nii's performance. I have tried to design this software to be quick - it attempts to minimize the amount of time writing to disk (by using a large amount of RAM). The table below shows the time required to process a standard neuroimaging dataset (1060 DICOM images [325Mb] with 792 fMRI volumes [36 slices, saved as mosaics], one T1 weighted anatomical scan and a field map). This dataset is typical for a one hour scanning session. The table below shows the time (in seconds) for dcm2nii (and SPM5) to convert these images. The 'GZ' cells reflect times for creating FSL style compressed .nii.gz images, while the other cells report times for creating SPM5 style .hdr/.img pairs. In brief, creating uncompressed images is generally constrained by disk speeds, while creating compressed images is limited by your processing power.    |  |  |  |  |  |  |  | | --- | --- | --- | --- | --- | --- | --- | | Setup | eSata | Internal HD | USB HD | GZ eSata | GZ Internal HD | GZ USB HD | | dcm2nii 2166Mhz CoreDuo Laptop | 12 | 20 | 41 | 70 | 70 | 73 | | SPM5 2166Mhz CoreDuo Laptop | 94 | 138 | 165 | - | - | - | | dcm2nii 667Mhz G4 Laptop | - | 41 | - | - | 229 | - |   **Sample Datasets** Here are some sample images that help show whether images are converted with the correct image orientation:   1. GE DICOM dataset.     Twelve 4D EPI series, each with five volumes: s26692 axial ascending    sequentia, s26693: axial ascending interleaved, s26694: axial    descending sequentia, s26695: axial descending interleaved, s26696:    sagittal right to left sequential, s26697: sagittal right to left    interleaved, s26698: sagittal left to right sequential, s26699 sagittal    left to right interleaved, s26700: coronal P to A sequential, s26701:    coronal P to A interleaved, s26702: coronal A to P sequential, s26703:    EPI, coronal A to P interleaved. A water filled fiduical marker is    placed over the right temple of the participant. Data were acquired on    a GE-SignaHD-Excite scanner at 3 Tesla using an 8 Channel Brain Array    Coil. The first volume of each series has been marked with a '1' on the    image. (LMU Grosshadern) 2. Philips DICOM dataset.    Six 4D EPI volumes, each with two volumes: sagittal, coronal and axial    each with both 'ascending' and 'descending' slice order. The white line    added at the bottom should be shorter on the earlier volumes. (MUSC    Center for Advanced Imaging Research) 3. Philips PAR/REC dataset.     Five 4D EPI volumes - same raw data as Philips DICOM dataset, but only    a single axial volume is included.(MUSC Center for Advanced Imaging    Research) 4. Siemens Trio B12 DICOM dataset.     Six 4D EPI volumes, each with two volumes: sagittal, coronal and axial    each with both 'ascending' and 'descending' slice order. A saline bag    is placed near the participant's left temple. (USC McCausland Center) 5. Siemens Trio B13 DICOM DTI dataset.    From May-2007, dcm2nii attempts to generate FSL and medINRIA compatible    descriptions of the B-values (bval) and Diffusion Gradient Directions    (bvec). For instructions on using FSL and medINRIA, see my DTI page.    These sample images were simply designed to validate dcm2nii's    conversion: these protocols are not appropriate for any other use (our standard protocol is described here).    This large (33Mb) file includes four 20 direction EPI datasets: the    first two are true axial (aligned to scanner bore, not the    participant's head) and are identical except that the phase direction    is Anterior-Posterior in the first and Right-Left in second. The third    volume is identical to the second except that the imaging plane has    been rotated: a pitch and yaw have been applied. The final scan is a    coronal scan,also taken in plane with the scanner's bore with pahse    encoding in the right-left direction. The file also includes a matlab    function (dtivecs.m) that illustrates dcm2nii's calculation for    correcting the diffusion directions for the imaging plane, using    suggestions from dicom2ana (see below) and Paul Morgan. (USC McCausland    Center) 6. 24-artificially generated .nii.gz images branded with 'Left' and 'Right'    These images began life as a skull stipped version of the NIfTI-1 Test Data Set.    Then each volume was subjected to a different orthogonal rotation, including flips (e.g. reversing left-right dimension). This sample dataset is useful    for ensuring that your viewing software correctly reads the NIfTI coordinates. The images are provided in nii.gz format, but you can s    simply drag-and-drop these images onto dcm2niigui to convert them to uncompressed .nii or .hdr/.img files if you want to test    software that does not read compressed data.   **Alternatives** Each manufacturer has interpretted the DICOM data standard a bit differently. Therefore, you may want to test several programs to see which one is best suited for your data   1. LONI Debabeler is    a Java applet that can run on just about any computer. It can also read    a number of medical imaging formats. Another nice feature is that it    reorients the raw data to be approximately aligned with the nearest    orthogonal orientation (i.e. coronal and sagittal scans are resliced    along the axial plane). 2. SPM5 includes a DICOM to NIfTI covnerter that works particularly well for Siemens data (requires Matlab). 3. dicom2nifti is a Matlab script for converting DICOM to NIfTI (requires Matlab and the Matlab Image Processing Toolbox).    [an alternative version is described here.] 4. xmedcon offers limited NIfTI writing support for many image formats. It uses the niftilib tools, which look very useful. 5. MRIconvert is a popular converter for Windows and Linux. 6. dinifti looks useful. 7. Here is a script that uses dicom2 and FSL to convert DICOM images to NIfTI. 8. XMedCon    includes the ability to convert between Acr/Nema 2.0, Analyze (SPM),    Concorde/µPET, DICOM 3.0, CTI ECAT 6/7, NIfTI-1, InterFile3.3 and    PNG or Gif87a/89a formats, as well as an elegant image viewer. 9. r2aGUI    Converts Philips PAR/REC images to NIfTI. Requires Matlab. 10. MIView     is an elegant medical image viewer for Windows. You can use the Tools/ConvertFiles command to convert DICOM images to NIfTI format. 11. The latest Philips Achieva research tools software can now directly produce (4D) nifti files This is a great alternative to using dcm2nii or r2agui. Kudos to Philips for directly supporting the NIfTI format. |

|  |
| --- |
|  |
