# Supplementary material for: Disturbed neurovascular coupling in hemodialysis patients
Source: PeerJ. 2020 Apr 15;8:e8989. doi: 10.7717/peerj.8989 (PMC7166048; doi:10.7717/peerj.8989)
Supplement: Supplemental Information 1 — Data imagings (T map), subjects and clinical variables (CSV format) and software for nii format (mricron). [file peerj-08-8989-s001.zip › raw_data/software_for_nii_format/mricron/html/index.html]

MRIcron Index Page


|  |  |
| --- | --- |
|  | MRIcron Index |

|  |  |
| --- | --- |
| - Installation - Putting this software on your computer. - Introduction - Basic usage, showing statistical overlays, rendering - **dcm2nii** - Converting scanner images (DICOM) to NIfTI format.   Allows programs like MRIcron, FSL and SPM5 to view scans. - Statistics - lesion symptom mapping - Peristimulus plots - viewing fMRI datasets - Batch and initialization Files - Advanced tips and tricks |  |

|  |
| --- |
|  |
