# Supplementary material for: Disturbed neurovascular coupling in hemodialysis patients
Source: PeerJ. 2020 Apr 15;8:e8989. doi: 10.7717/peerj.8989 (PMC7166048; doi:10.7717/peerj.8989)
Supplement: Supplemental Information 1 — Data imagings (T map), subjects and clinical variables (CSV format) and software for nii format (mricron). [file peerj-08-8989-s001.zip › raw_data/software_for_nii_format/mricron/html/install.html]

MRIcron Installation Page


|  |  |
| --- | --- |
|  | MRIcron Installation |

**Introduction :: Current version is 12/2009**

MRIcron can run on Windows, Linux and Macintosh OSX computers. Please select the version that works best for your operating system.

- Windows (WinAPI widgetset)
- Linux

- 32-bit (GTK1 widgetset)
- 32-bit (GTK2 widgetset)
- 64-bit (GTK1 widgetset)
- 64-bit (GTK2 widgetset)

- Macintosh OSX

- OSX 10.4 or later (Universal binary, Carbon widgetset)
- OSX 10.3 or earlier (PowerPC, GTK1 widgetset)

**License**

Chris Rorden's MRIcron, copyright 2007, all rights reserved. Redistribution and use in binary forms, with or without modification,
are permitted provided inclusion of the copyright notice, this
list of conditions and the following disclaimer is provided with the
distribution:  Neither the name of the copyright owner nor the name of
this project (MRIcron) may be used to endorse or promote products
derived from this software without specific prior written permission.This software is provided by the copyright holder "as is" and any express or implied warranties, including, but not limited to, the implied warranties of merchantability and fitness for a particular purpose are disclaimed. In no event shall the copyright owner be liable for any direct, indirect, incidental, special, exemplary, or consequential damages (including, but not limited to, procurement of substitute goods or services; loss of use, data, or profits; or business interruption) however caused and on any theory of liability, whether in contract, strict liability, or tort (including negligence or otherwise) arising in any way out of the use of this software, even if advised of the possibility of such damage.

**Windows** [Requires Windows 95 or later; works on both 32-bit and 64-bit versions of Windows]

1. Download the installer.
2. Unzip and run the installer (double click on the .zip file and run the program InstallMR.exe).
3. By default, the installer places the programs in C:\program
   files\mricron, simply double click on mricron.exe to run the software.
4. You may also double-click on the .bat files, which load example
   images, for example 'examplefmri.bat' shows two statistical maps on top
   of a high resolution anatomical scan.

  

**Linux x86 32bit GTK1 Note that it is easier to install the GTK2 version (see below) with recent (>2005) distributions of Linux.** 

1. Download the software.
2. Unzip the software (for modern Linux systems, right click on the file mricronlx.zip and choose to 'extract here').
3. Open a terminal window.
4. Change to the folder where you installed mricron, e.g. cd ~/mricron
5. Launch mricron by typing ./mricron (note you must be in the same folder as the mricron executable.
6. You can also run the example .bat files, for example ./xfmri.bat
   shows two statistical maps on top of a high resolution anatomical scan.

1. In case you receive error messages, check if all the dependencies of the program are installed. In particular, you need

1. GTK 1.2 (this is called GTK+ by some distributions, and gtk by others)
2. libgdk\_lixbuf2.0

2. You can see a complete list of .so files needed by the software using the command:  
     ldd ./mricron
3. In case you cannot run the program and it displays a message
   saying it cannot find one of these libraries (you must run the program
   from the command line to see error messages), you need to download and
   install a package for the necessary library:

   - On RPM-based distributions you can find packages here: http://rpm.pbone.net/
   - On Debian-based distributions you can find packages here: http://www.debian.org/distrib/packages

  

**Linux x86 32bit GTK2 Note that the GTK1 version (above) has been more extensively tested.**

1. Download the software.
2. Unzip the software (for modern Linux systems, right click on the file mricronlx2.zip and choose to 'extract here').
3. Double-click on the mricron application icon to launch the program.

  

**Linux x86 64bit GTK1** 

1. Download the software.
2. Unzip the software (for modern Linux systems, right click on the file mricronx641.zip and choose to 'extract here').
3. Double-click on the mricron application icon to launch the program.

  

**Linux x86 64bit GTK2** 

1. Download the software.
2. Unzip the software (for modern Linux systems, right click on the file mricronx64.zip and choose to 'extract here').
3. Double-click on the mricron application icon to launch the program.

  

Macintosh Intel **[Universal Binary]**

1. Download the software.
2. Unzip the software (double click on the .zip file - a new folder called mricron will be created).- **Optional:** If you are using OSX 10.5 or later, you may want to consider the DTI-TK NIfTI Quick Look Plugin. This will preview NIfTI images from the finder and the open dialog box.

  

Macintosh PowerPC  **OSX 10.4 or later [Universal Binary]**

1. Download the software.
2. Unzip the software (double click on the .zip file - a new folder called mricron will be created).

  

Macintosh PowerPC GTK **OSX 10.3 or earlier**
  
 **Note:**Users with OSX 10.4 or later should run the standard "Macintosh PowerPC" version of MRIcron. This version of MRIcron for the Macintosh PowerPC requires GTK to be installed. In many cases the best solution is to run the Windows-native version of MRIcron using VirtualPC.

1. Download the software.
2. Unzip the software (double click on the .zip file - a new folder called mricron will be created).
3. You will need to install X11.

1. Check to see if X11 is in the applications\utilities folder.
2. If X11 is not installed:

1. OSX 10.4 user can install X11 by using the DVD installer disk
2. Alternatively, download and run the X11 installer distributed by Apple.

4. Download and install the correct version of fink
   for your version of OS X and let it download and install the additional
   29 GTK and related packages needed. Here's the required command for my
   software (all on one line):

- sudo fink install gdk-pixbuf gtk+ gtk+-data gtk+-shlibs gtk-doc gtkglarea gtk-engines

5. Launch X11 (double click on the X11 icon in the applications\utilitiesfolder).
6. Launch an X11 terminal  (from the keyboard, type Apple-N). From the terminal you can do the following

1. Change to the folder where you installed mricron, e.g. cd ~/Documents/mricron
2. Launch mricron by typing ./mricron (note you must be in the same folder as the mricron executable.
3. You can also run the example .bat files, for example
   ./xfmri.bat shows two statistical maps on top of a high resolution
   anatomical scan.

  
  

Source Code  [All platforms]

1. Install the  Lazarus IDE with Freepascal compiler. You may want to download one of the latest builds.
2. Get the source code. The source code is available here.
3. You can build the software from the command line (see the 'script' files in the MRIcron folder, these use the command lazbuild, for example "lazbuild ./mricron.lpr --ws=carbon" compiles the software for the OSX Carbon widgetset). Alternatively, you can build from the IDE (launch the Lazarus program and choose File/Open to select the project file [.lpr extension]).

|  |
| --- |
|  |
