# Supplementary material for: Disturbed neurovascular coupling in hemodialysis patients
Source: PeerJ. 2020 Apr 15;8:e8989. doi: 10.7717/peerj.8989 (PMC7166048; doi:10.7717/peerj.8989)
Supplement: Supplemental Information 1 — Data imagings (T map), subjects and clinical variables (CSV format) and software for nii format (mricron). [file peerj-08-8989-s001.zip › raw_data/software_for_nii_format/mricron/html/main.html]

MRIcron Introduction Page


|  |  |
| --- | --- |
|  | MRIcron Introduction |

**Main Window**

I have tried to make the MRIcron main window as useful but simple as
possible. While the options may appear confusing at first, I hope over
time the software feels intuitive. A sample window is shown below. You
can get a similar view by launching the software, choosing
File/OpenTemplates/ch2bet to load the background image (shown as a
grayscale brain) and then choosing Overlay/Add and opening the image
\Template\attention.

the controls on the tool bar offer quick access to the main tools
for brain imaging. The X/Y/Z numbers set the slice to view (X refers to
Left/Right, Y to anterior/posterior and Z to superior/inferior).
Adjusting these values will change the sagittal, coronal and axial
slice displayed. The next item is the zoom-factor. Here images are
scaled 'to fit' - e.g. each view of the brain is strecthed to fill its
panel optimally. Alternatively, you could choose x1 (100%), x2 (200%)
or x3 (300%) zoom factors. The next series of buttons refer to the
active layer (more on layers in the next section). In the example
below, the currently active layer is image 'attention', and this image
is being shown as a red gradient with a minimum value of 1.96 to 4. In
this example, attention is a statistical Z-score map that has been
placed on top of the background image of a brain. Statistical values
less than 1.96 are not shown, with the darkest value set to 4.

|  |
| --- |
| This image shows a sample rendering. You can view a similar image by running the fmri.bat file that is included with MRIcron. |

Layers

When you use the File menu's Open command (Open, Open recent and
Open Templates) you are selecting a background image. What makes
MRIcron powerful is its ability to load multiple layers of images. To
add additional images on top of your background, simply choose 'Add'
from the 'Overlay' menu. For example, launch MRIcron and choose
'File\Open templates\ch2bet', then choose 'Overlay/Add' and select the
'attention.nii.gz' image from the 'Example' folder that (typically,
this folder is installed in C:\program files\mricron\examples). Now you
should see a brain activation statistical map on top of the background
anatomical image. Note that overlay images are scaled to map on top of
the background image. You can adjust the appearance of each layer by
using the layer panel:

Note that you can click on the leftmost button in the overlay panel to
select between the open layers - for example the image above shows
settings for the 'Background' image, as well as the overlay image named
'saccades'. Note that the image intensity range for the background is
from 45 to 120, using a grayscale color scheme. This means that values
of 45 or lower will appear as complete black, and 120 and above will be
white, with intermediate intensities appearing as a corresponding gray
value. There is also an icon with a color scale and the number zero:
this allows you to have the color range set from zero, even if your
threshold is greater than zero: for example our statistical map
'saccades' is set to show values from 2.3 to 6: values less than 2.3
will not be shown. However, since the 'color range from zero' button is
depressed, a T-score of 2.3 will appear as a dark red (as 2.3 is part
way between 0 and 6) instead of a black (if the color range was from
2.3 to 6).

MRIcron allows you to choose different color schemes for each layer. In the example above, the Background image is shown using the
'grayscale' black-and-white colorscheme, while the saccade overlay uses the Red color scheme. You can increase or decrease the
colorschemes available by adding or removing files from the 'LUT' folder (this 'look up table' folder is located in the same path as MRIcron).
This folder typically contains several \*.lut files, with each file storing a different color scheme. For example, the blackbdy.lut' file describes a color scheme that goes from black to orange to yellow and finally white. MRIcron's \*.lut files are interchangeable with the \*.lut files used by ImageJ, XMedCon, MRIcro. You can copy \*.lut files from these programs to MRIcron's LUT folder. Furthermore, you can create your own \*.LUT files using ImageJ's LUT panel plugin, ImageJ's LUT Editor or my own LUTmaker.

Note that the 'Overlay' menu also allows you to modify how layers
appear. For example, on image 'A' below, I have selected
Overlay/TransparencyOnBackground/0%[Opaque], while for image B, I have
set the transparency to 50%. Also note that you can load multiple
overlays simultaneously. For example, images C and D below show the
'attention'  and  'saccades' files both overlayed on top of
ch2bet - with attention shown in green and the saccades shown as red.
For image C, I have set the Overlay/TransparencyOnOtherOverlays/50%,
while for Figure D this has been set to 'Additive' (so regions with
both red and green appear yellow).

**Render Window**

|  |  |
| --- | --- |
| From MRIcron's main window you can select Window/Render to view a volume rendering of your data. You can change the vieiwing angle of the image by adjusting the Aizmuth and Elevation values. The Background menu allows you to adjust therendering of the background image, while the background menu allows you to determine how overlays will appear. Of critical importance, the Overlay/SearchDepth will adjust whether the rendering shows superficial overlays (e.g. a settings of 4 will only look within the first 4 voxels of the background image [e.g. within the first 4mm if your background image hasa 1mm resoultion), while a setting of 'infinite' will show an overlay regardless of its depth. Also criticial is the Background/BehindOverlay menu item - if checked, the software will only start looking for overlays that exist behind the surface of the background. On the other hand, if this value, overlays will not be constrained by the background image (e.g. you will be able to view an overlay that is closer than the background). You can save your preferred settings by selecting File/SaveSettings. This information will be saved in the 'render' folder, and you will be able to select them by choosing File/OpenSettings. | This image shows a sample rendering. You can view this image by running the fmri.bat file that is included with MRIcron. |

**Multislice Window**

From MRIcron's main window you can select Window/Multislice to
see the multislice window. You will then be shown a series of slices of
the currently open volumes. Within the Multislice form you can use the
View meanu to adjust settings - e.g. to select whether you want to see
sagittal, coronal or axial images, and to choose the desired slices and
overlap between neighboring slices. You can also choose
File/SaveSettings to save your favorite views (previous sets will be
listed under File/OpenSettings).

|  |  |
| --- | --- |
| This image shows a sample multslice image. You can view this image by running the fmri.bat file that is included with MRIcron. After MRIcron loads, choose Window/Multislice. Note you can adjust the amount of overlap between slices, as well as slice orientation. Forthermore, you can choose which slices to display. |  |

  

|  |
| --- |
|  |

  
