# Supplementary material for: Disturbed neurovascular coupling in hemodialysis patients
Source: PeerJ. 2020 Apr 15;8:e8989. doi: 10.7717/peerj.8989 (PMC7166048; doi:10.7717/peerj.8989)
Supplement: Supplemental Information 1 — Data imagings (T map), subjects and clinical variables (CSV format) and software for nii format (mricron). [file peerj-08-8989-s001.zip › raw_data/software_for_nii_format/mricron/html/peri/index.html]

MRIcron Peristimulus Plots


|  |  |
| --- | --- |
|  | Peristimulus Plots |

**Introduction**

SPM and FSL are powerful tools for analyzing fMRI data. However, the
statistical maps most people generate with these tools can be difficult
to interpret. Generating peristimulus plots can allow you to get a
better idea of what your data actually looks like, and can help you
determine if a region shows an increased amplitude of activity or a more
sustained response to a stimuli. To generate peristimulus plots you
will need:  

1. MRIcron.
2. A 4D fMRI dataset (typically motion corrected and smoothed)
3. A FSL format 3-column text file for each condition you wish to analyze.
4. Optional: regions of interest for specific brain regions.

You can also click here to download a sample data set (14mb).  

**Basic Usage**

Here are step-by-step instructions  

1. launching MRIcron. Then choose '4D traces' from the View menu.
2. Press the 'Open Data' button.

- You will be asked to load a sample dataset, choose filtered\_func\_data.nii.gz
- Optional: You will be asked to load event onset files, choose both L\_Tap.txt and R\_Tap.txt
- Optional: You will be
  asked whether you want to load any regions of interest. These can be
  hdr/img; nii; or voi files (note you have to pull down the file-type
  menu to select voi files). Select L.voi and R.voi

3. MRIcron will now display a timeline for your data. If you have
   loaded multiple regions, a separate line displays each ROI. If you have
   not selected any ROIs, you will be shown the currently selected voxel -
   use MRIcron's main window to select a voxel you want to view and then
   press the red refresh button in the timeline window to see the
   timeiline for this voxel. Note that if you have loaded any event
   onsets, each condition is shown as a unique color of vertical stripes -
   for example in the example left hand taps are shown as red bars and
   right taps are shown as green bars. Note with the example datasets that
   left taps are followed by increases in signal for the right ROI, while
   right taps are followed by increasing signal in the left hemisphere.
4. Before generating peristimulus plots, make sure that the TR is
   accurately set. Our sample data has a TR of 3 seconds, and this is
   correctly reported in the image file, so MRIcron correctly reports a TR
   of 3 seconds. If your TR is incorrect, the events will not be correctly
   aligned with your images.
5. Press the 'Plot' button to generate phase-locked peristimulus
   plot. You will want to check the settings for your peristimulus plot

1. The bin width sets the resolution for plot - smaller bins are
   more precise but noiser. By default, the bin width is set to your TR,
   in our example 3 seconds.
2. The pre-stimulus bins sets the number of baseline bins. In our example we are setting 4 bins (12 seconds).
3. The number of post-stimulus bins plot signal changes after an
   event has been presented. Remember that fMRI signals are sluggish, and
   take 5-6 seconds to peak. For the example, set this to 14 (42 seconds).
4. If you slice time corrected your data, check the appropriate
   box. Event times will be adjusted for the acquisition of the
   middle-slice in your volume (e.g. all of your onsets will be adjusted
   by 0.5 TR).
5. The save peristimulus volume button allows you to save a
   separate 3D dataset for each time bin. This is an advanced feature we
   will discuss later.

6. MRIcron generates a peristimulus plot. Different colors are used
   for the different conditions, while different line styles are used for
   the different regions of interest. For our example, note that the right
   hemisphere shows a response for left but not right taps, while the
   reverse is true for the left hemisphere. The peak amplitude is about 1%
   signal change. While this effect sounds small, note that we are
   averaging over a large number of voxels which in this case were
   selected baseed solely on anatomy, rather than post-hoc selecting the
   single most active voxel. Also note that the error bars are rather
   small.

  
  

**Removing Regressors**

The recent versions of MRIcron (since December, 2007) allow you to remove the influence of other conditions. This feature can remove expected but uninteresting variance in your data, resulting in a better signal to noise. The images below illustrate how this works. Consider an event related design where an individual occasionally taps her left hand, and at other times she taps her right hand. If we looked at the motor cortex of the left hemisphere (which predominantly controls the individual's right hand) our data might look like this:  
  
The observed signal is actually a combination of brain activity due to movement of the right hand, somewhat weaker activity following movement of the left hand and some noise. Since we know when the individual moved their hand, and we know the approximate pattern of the hemodynamic response, we can use multiple linear regression to estimate relatively pure signals of left hand movement and right handmovement, as shown here:  
  
This can help visualize our peristimulus plots. If we were to make a peristimulus plot of the raw data for the left hand movement (top panel of the image below) the observed data would have an unusual latency and a lot of noise, because this plot includes not only responses for the left hand but also noise from the brain activation following movement of the right hand. The lower plot shows the effect of removing the irrelevant regressors. Note that this reveals a much purer effect. By default, MRIcron will plot the observed data after regression (e.g. a mixture of the signal of interest plus noise). However, you can alternatively choose to see the 'modeled rather than observed data'. This will show you the convolved effect of interest, with the amplitude estimated by the multiple regression. This is useful for seeing if the observed data has roughly the same latency as predicted by the HRF. If this differs a little bit (<1 sec), you will want to include temporal derivatives in your model. If the latency is dramatically different, you should check to ensure that your onset file is correct and consider using a tool like
FLOBS to analyze your data.
  

**Advanced Usage**

- The current version of my software uses the following algorithm to generate peristimulus plots. This algorithm has a direct impact on the results, and may not be suitable for all types of data:

- Each observation (e.g. each of the 4D volumes) will only influence a single bin. In other words, a volume that is acquired 8 seconds after event 1, 2 seconds after event 2 and 3 seconds before event number 3 will only contribute to a single bin (e.g. the bin that for events that occur 2 seconds after an event).
- An observation will influence the bin that it most closely **follows**. In other words, a volume that is acquired 8 seconds after event 1, and 2 seconds after event 2 will only contribute to a single bin (e.g. the bin that for events that occur 2 seconds after an event). This is a common procedure, but note that with the sluggish HRF it is not really suitable for situations where events occur VERY rapidly (e.g. the HRF is sluggish, and takes about 5 sec to peak, therefore an event that occurred 5 sec ago will have a stronger influence than one that occurred 2 seconds ago).
- An observation will only be included in a pre-stimulus bin if it can not contribute to any post-stimulus bins. In other words, if your peristimulus plot covers a period from -6 seconds to +12 seconds, a observation will only contribute to the -6..0sec bins if at least 12 seconds elapsed since the previous event. This is important, as we do not remove variability associated with the signal of interest, therefore a stimuli that occurs 6 sec after an event but 1 second before the next event is likely to show a bright intensity from the earlier event.

- For statisitcal analysis, you will want the precise values for
  the effect sizes and standard errors. If you press the 'text' button
  instead of the plot button, you will be shown the precise values - save
  these as a .csv (comma separated values) format file to open them and
  generate nice graphs with Excel or other software.
- You can save peristimulus volumes to see precisely what is
  happening for each voxel at a given time point. The image below shows
  the 8th time bin for both the left and right movement conditions. Note the
  bright contralateral signal.
- When generating peristimulus plots, you have an option of choosing whether your data has been slice time corrected or not. This will influence how the event times are interpretted. Without STC, my software assumes that the event times are relative to the first slice of the first volume. On the other hand, if STC is checked, all times are relative to the acquisition of the middle slice of the first volume. The image below illustrates this (assuming a 2 second TR): without STC, the first event occurs at 0.5sec, and the second event occurs at 6 seconds. With STC, the first event occurs at -0.5s, and the second event occurs at 5s. Sparse acquisition is useful for auditory studies (as stimuli can be presented while the scanner is silent, and we can then observe the sluggish consequences of this). However, most sparse studies will not yield good peristimulus plots (good plots will required jittered stimulus-scan intervals). In any case, for sparse imaging I suggest making sure STC is NOT selected, and having the origin for event times to be the acquisition of the irst slice. Note that my software does not adjust for differences in slice time acquisition within a volume. The whole 3D volume is assumed to have been collected at a single instant. Therefore, (for axial acquisitions) signals from the cerebellum will appear to have a different lag than signals from the top of the brain. If you want to make comparisons between different brain areas, you may want to slice time correct your data before generating peristimulus plots.

  
Notes   
  
Russ Poldrack's
ideas inspired many features in my software.  
MarsBar
is a popular region of interest analysis tool.  
Featquery
is useful for investigating FSL findings.  

|  |
| --- |
|  |
