# Supplementary material for: Disturbed neurovascular coupling in hemodialysis patients
Source: PeerJ. 2020 Apr 15;8:e8989. doi: 10.7717/peerj.8989 (PMC7166048; doi:10.7717/peerj.8989)
Supplement: Supplemental Information 1 — Data imagings (T map), subjects and clinical variables (CSV format) and software for nii format (mricron). [file peerj-08-8989-s001.zip › raw_data/software_for_nii_format/mricron/html/source.html]

MRIcron Source Code Page


|  |  |
| --- | --- |
|  | MRIcron source code |

Introduction

MRIcron is written in Pascal, and can be compiled using Borland's
Delphi or the open source Lazarus (Freepascal) software. MRIcron is open source, using the BSD license. Lazarus offers
several advantages - it is free, comes complete with all the components
required by MRIcron, and offers the potential to easily create native
binary code for Windows, Linux, and OSX. However, Lazarus is still beta
software.

1. Install the compiler. You will need Lazarus version 0.9.24 or later. This software requires some recent patches that
   are not available on the current stable release.
2. Get the source code. The source code is available here. The source
   file includes sample images in the Templates and Example folders. To
   test this software, compile mricron.lpr. Next use the File/templates
   menu to open sample images. Alternatively, after compiling the
   software, you can click on the included .bat files to see sample
   renderings (see the included readme.txt for more information, especially for OSX compilation).

|  |
| --- |
|  |
