# Supplementary material for: Disturbed neurovascular coupling in hemodialysis patients
Source: PeerJ. 2020 Apr 15;8:e8989. doi: 10.7717/peerj.8989 (PMC7166048; doi:10.7717/peerj.8989)
Supplement: Supplemental Information 1 — Data imagings (T map), subjects and clinical variables (CSV format) and software for nii format (mricron). [file peerj-08-8989-s001.zip › raw_data/software_for_nii_format/mricron/html/stats.html]

MRIcron Statistics Page


|  |  |  |
| --- | --- | --- |
| |  |  | | --- | --- | |  | MRIcron Statistical Analysis Tutorial | |

|  |  |  |  |  |  |  |  |  |  |  |  |  |  |  |  |  |  |  |  |  |  |  |  |  |  |  |  |  |  |  |  |  |  |  |  |  |  |  |  |  |  |  |  |  |  |  |  |  |  |  |  |  |  |  |  |  |  |  |
| --- | --- | --- | --- | --- | --- | --- | --- | --- | --- | --- | --- | --- | --- | --- | --- | --- | --- | --- | --- | --- | --- | --- | --- | --- | --- | --- | --- | --- | --- | --- | --- | --- | --- | --- | --- | --- | --- | --- | --- | --- | --- | --- | --- | --- | --- | --- | --- | --- | --- | --- | --- | --- | --- | --- | --- | --- | --- | --- |
|  | **Introduction** **Important note: By default, MRIcron has the lesion drawing tools switched off. To turn on the lesion drawing features of MRIcron, select Help/Preferences and make sure the "Show drawing menu and tools" checkbox is selected.**  MRIcron is designed to relate lesion location to behavioral performance. For example, it can help identify brain regions that are crucial to language production. To conduct an analysis, we will need to conduct four steps:   1. Lesion Mapping: For each individual, we need to map the extent of brain injury. 2. Specify design: We need to design our experiment, creating a    spreadsheet that links each individual's lesion map to their performance 3. Compute results: We need to conduct a voxelwise statistical analysis. 4. Viewing results: We need to interpret the results.  This tutorial guides you through a lesion data analysis.    1. A copy of MRIcron: the version will include a folder named 'example\lesions' with the sample dataset described here. 2. A copy of NPM (installed when you install MRIcron)  In this tutorial, I assume the lesion maps and design file are in the folder c:\dataset, but you can extract the files anywhere. Note that by default these are usually installed to c:\program files\mricron\example\lesions. The sample dataset includes simulated lesion maps for 23 patients. This folder also includes .val files that report the performance of these patients on a letter cancellation task.  In this task, patients are asked to mark each occurence of the letter 'A' on a piece of paper that was cluttered with letters. A perfect score on this task is 60 (when all the A's are detected). The file continuous.val lists each patient's performance on this task (a score of 2..60), while the file binomial.val lists performance on this task as binary: patients missing more than 4 items are listed as having failed this task (0), while patients who missed 4 or fewer items are listed as having passed this task (1). **Note that for both the continuous and binomial measures, a higher score indicates BETTER performance. If lower scores indicate better performance (e.g. response times) you need to either look at the negative Z-values in the statistical maps or invert the magnitude of your behavioral data.** We have included both binomial and continuous values to illustrate the statistical analyses available with MRIcron.     |  |  |  | | --- | --- | --- | | Right: A sample lesion map (9.voi) overlayed ontop of the ch2 template showing injury to the left temporal lobe. To view this map, launch MRIcron and choose File/Template/Ch2, then choose Overlay/Add... and choose the image 9.voi included with the sample dataset. |  |  |   **Lesion Mapping**  MRIcron provides simple tools for drawing a region of brain injury. However, it is crucial that all of our lesion maps are drawn with the same image dimensions and orientation. Therefore, we should either draw all the lesions on a standard template (e.g. File/OpenTemplates/CH2), or we need to first normalize all the scans so they are coregistered and then open each scan using File/Open.   1. Launch MRIcron and open your scan (File/Open or File/OpenTemplate) 2. Select your drawing tool (these are listed at the bottom of the Draw menu, e.g. the 'Pen' tool). 3. Draw your region - for example if you use the 'Autoclose Pen'    tool, simply click and draw the border of the brain injury. To fill in    an enclosed region, simply shift+click in the center of the region. To    erase part of your drawing, hold down the Shift key. 4. Repeat step 3 for all slices where a lesion is present (e.g. you    can adjust the X,Y,Z numbers that appear on the top left to select the    desired slice. Note you can also use a mouse scroll-wheel to select    slices. 5. When you are done drawing the region of brain injury, choose Draw/SaveVOI to save a copy of the lesion map. 6. Repeat steps 1-5 for each individual, save the lesion maps from all the individuals in a single folder.     |  |  |  |  |  |  | | --- | --- | --- | --- | --- | --- | |  |  |  |  |  |  | |  | Pen Tool | Closed Pen Tool | Fill Tool | Circle Tool | 3D Fill Tool | | Left click | Draw line | Draw closed line | Fill region | Draw ellipse | see web page | | Shift+ left click | Erase line | Erase closed line | Erase region | Erase ellipse |  | | Ctrl+ left click | Draw thick line | Draw thick closed line | 3D Bubble Fill | Draw rectangle |  | | Ctrl+shift+ left click | Erase thick line | Erase thick closed line | Erase 3D region | Erase rectangle |  | | right click | Fill region | Fill region | Fill region | - |  | | Shift+ right click | Erase region | Erase region | Erase region | - |  | | Alt+ left click | Change view | Change view | Change view | Change view | Change view |   Specify the Design Lets famialize ourselves with the dataset we will analyze.  1. Launch NPM and open the design window    (VLSM/Design...). A speadsheet will appear. Select File/Open    and view the file continuous.val. Each row shows    the performance of each patient, for example the patient 1    identified 2 items, while patient 2 detected 44 items. Note that with    this software higher scores reflect better    performance. If you have binomial data (where performance falls into    two discrete categoris), you should denote the presence of a deficit    with a 0 and healthy performance with a one. Note that the filename    listed in the left column and the performance in the right column    always correspond to the same patient. 2. We need to first describe the lesion maps and name the behavioral    performance measures. Select View/Design to bring up the description    window.           1. Predictors: shows the number of behavioral measures - here we are only examining the letter cancelation performance..    2. Predictor names: for each predictor, insert an easy to remember name, e.g. 'cancel' for our letter finding task.    3. You can select the file names of your lesion maps by       pressing the 'Select Images' button to select the lesion maps from       all your participants. You should select all the images simultaneously,       and all the images should be placed in a single folder (e.g. your       lesion maps might be C:\dataset\1.voi, C:\dataset\2.voi, etc). The lesion       images can be in MRIcron VOI, NIfTI .nii, compressed NIfTI .nii.gz or       Analyze (.hdr/.img) format. If you do select images, make sure the       filenames match the patient performance, as noted in step 1.    4. You can also set an a priori minimum lesion density threshold. For       example, setting a value of 10% when you analyze 22 people means that       statistics will only be computed for voxels damaged in more than 2       people. A large number for this threshold can increase your statistical       power, as you will only compute statistics for voxels that are commonly       injured. However, larger values will fail to detect rarely damaged       regions that are reliable predictors of deficit. Note that this value       is based on the total incidence of lesions in a voxel, regardless of       behavioral performance.   **Compute results**  Next, we we compute our statistical results. You can conduct some statistics by choosing items in the Draw/Statistics menu of MRIcron. However, here we describe using some of the new features in NPM that are not yet available in MRIcron - specifically, NPM can conduct permutation thresholding and the Brunner and Munzel test. To conduct these statistics, you need to download and install npm.exe - this only works on the Windows operating system.   1. First go to the 'Options' menu and set the permutations to None.    Permutation thresholding can be useful, but it will take at least 1000    times longer than a normal False-Discovery Rate corrected threshold,    and is typically less sensitive. Therfore, for you first glance at your    data, turn this feature off. 2. For analyzing the continuous data:  1. Go to 'Option' menu and click 'Tests' - make sure the 'Brunner Munzel' is checked and the t-test is unchecked.    Our data is not normally distributed, so we will use a non-parametric    test (using the permuted Brunner Munzel rank order statistic). 2. Click the VLSM/BinaryImagesContinuousGroups command. Select the continuous.val file.  3. For analyzing the binomial data:  1. Click the VLSM/BinaryImagesBinaryGroups command. Select    the binomial.val file. This will use the Liebermeister measure (a more    sensitive binomial test than Chi-Squared or Fisher's Exact test, see Seneta and Phipps, 2001; Phipps, 2003).  4. NPM will now compute the requested tests. It will create    overlap images of all your patients (e.g. sum.nii.gz) and a statistical    map (BM.nii.gz for continuous data, L.nii.gz for binomial data).   **Viewing results**  We can open up the statistical maps generated and place them on top of an anatomical scan. If your lesion maps were aligned to stereotaxic MNI space, you can open them on top of one of the standard templates (File/OpenTemplates/ch2). Here is a quick guide:   1. Launch MRIcron and choose File/OpenTemplates/ch2bet as our background image 2. Choose Overlay/Add and choose the statistical map created in the previous step (e.g. C:\dataset\binL.nii.gz). 3. When MRIcron detects a statistical map, it calculates the    p-values    for each test in order to determine the false discover rate (FDR)    threshold - e.g. how much robust signal is present in your data.    MRIcron displays a histogram of the Z-scores. Note in the example    below, most of the data has positive Z scores suggesting a robust    signal (if our data was merely noise, we should see a bell-shaped    distribution with a mean of 0, instead the mean Z score is around 2). 4. Next, MRIcron displays the overlay. Note at the bottom of the    screen the software reports the critical values: the p05/p01 values    correspond to uncorrected p<0.05 and p<0.01 values that are very    liberal (these tests will make many false alarms, e.g. here we have    conducted 16082 tests, so p05 should result in many false positives).    The fwe05 and fwe01 values correspond to the Bonferroni-corrected    values: this test is very conservative, and you will often fail to    detect real effects. The FDR05 and FDR01 results reflect the False    Discover Rate - e.g. a FDR05 should show around 20 real activations for    every false positive. Note that when there is very little or no signal,    FDR is as conservative as Bonferroni, but it is adaptive to the actual    signal in your dataset. Note that you can select the image thresholding    and cutoff for your overlay. Note that by default,    my software loads statistical maps with thresholds from FDR05 to FDR01,    unless there is insufficient signal, in which case it uses the    uncorrected 0.05...0.01 values. Also note that the current overlay is    set to appear in a monochromatic red color scheme.          The image below shows how changing a threshold can change the    appearance of a statistical overlay. Consider the raw statitical map    shown in the left panel, while the middle panel has been thresholded to    only show voxels with Z-scores greater than 2.5 (with three regions    surviving this threshold), the right panel shows a more conservative    threshold of Z>4.5 - with only a single peak surviving. 5. We can repeat steps 2..4 to load multiple overlays, to compare    different statistical tests. For example, clicking on the    'tutorialfmri.bat' icon will launch MRIcron and load to overlapping    regions of interest. By using the Overlay/TransparencyOnOtherOverlays    command we can view both of these overlays simultaneously. |

|  |
| --- |
|  |
