# Supplementary material for: Disturbed neurovascular coupling in hemodialysis patients
Source: PeerJ. 2020 Apr 15;8:e8989. doi: 10.7717/peerj.8989 (PMC7166048; doi:10.7717/peerj.8989)
Supplement: Supplemental Information 1 — Data imagings (T map), subjects and clinical variables (CSV format) and software for nii format (mricron). [file peerj-08-8989-s001.zip › raw_data/software_for_nii_format/mricron/html/tutorial/mricrondata.zip!/index.html]

MRIcron Statistics Page


|  |  |
| --- | --- |
|  | MRIcron Statistical Analysis Tutorial |

**Introduction**

Important note: By
default, MRIcron has the lesion mapping tools switched off. This is an
advanced feature, and is still being developed. To turn on the lesion
drawing features of MRIcron, you will need to edit the mricron.ini with
a text editor - you need to change "ShowDraw=0" to "ShowDraw=1".

This tutorial guides you through a lesion data analysis.   

1. A copy of MRIcron (version 9/9/2006 or later). www.mricro.com/mricron/
2. A copy of NPM.exe for Windows (version 9/9/2006 or later) with the sample dataset

In this tutorial, I assume these files are
extracted to the folder c:\dataset, but you can extract the files
anywhere. The sample dataset includes simulated lesion maps for 23
patients. This folder also includes .val files that report the
performance
of these patients on a letter cancellation task.  In this task,
patients are asked to mark each occurence of the letter 'A' on a piece
of paper that was cluttered with letters. A perfect score on this task
is 60 (when all the A's are detected). The file continuous.val lists
each patient's
performance on this task (a score of 2..60), while the file
binomial.val lists performance on this task as binary: patients missing
more than 4 items are listed as having failed this task (0), while
patients who missed 4 or fewer items are listed as having passed this
task (1). We have included both binomial and continuous values to
illustrate the statistical analyses available with MRIcron.  
  

|  |  |  |
| --- | --- | --- |
| Right: A sample lesion map (9.voi) overlayed ontop of the ch2 template showing injury to the left temporal lobe. To view this map, launch MRIcron and choose File/Template/Ch2, then choose Overlay/Add... and choose the image 9.voi included with the sample dataset. |  |  |

Examining the patient performance

Lets famialize ourselves with the dataset we will analyze.

1. Launch MRIcron and open the design window
   (Draw/Statistics/Design...). A speadsheet will appear. Select File/Open
   and view the file continuous.val. Each row shows
   the performance of each patient, for example the patient 1
   identified 2 items, while patient 2 detected 44 items. Note that with
   this software higher scores reflect better
   performance. If you have binomial data (where performance falls into
   two discrete categoris), you should denote the presence of a deficit
   with a 0 and healthy performance with a one. Note that the filename
   listed in the left column and the performance in the right column
   always correspond to the same patient.
2. We need to first describe the lesion maps and name the behavioral
   performance measures. Select View/Design to bring up the description
   window.  
      
   1. Predictors: shows the number of behavioral measures - here we are only examining the letter cancelation performance..
   2. Predictor names: for each predictor, insert an easy to remember name, e.g. 'cancel' for our letter finding task.
   3. Template: This allows you to specify whether you want the
      statistical maps computed with a specific bounding box, e.g. you could
      choose an image with 2x2x2mm, or one with 1x1x1mm. If you do not
      describe a temple, the statistics will be mapped to your first
      participant. I generally suggest NOT specifying a template. To select a
      template, click on the 'select template' button and choose the image.
   4. You can select the file names of your lesion maps by
      pressing the 'Select Images' button to select the lesion maps from
      all your participants. You should select all the images simultaneously,
      and all the images should be placed in a single folder (e.g. your
      lesion maps might be C:\val\z1.voi, C:\val\z2.voi, etc). The lesion
      images can be in MRIcron VOI, NIfTI .nii, compressed NIfTI .nii.gz or
      Analyze (.hdr/.img) format. If you do select images, make sure the
      filenames match the patient performance, as noted in step 1.
   5. You can also set an a priori minimum lesion density threshold. For
      example, setting a value of 10% when you analyze 22 people means that
      statistics will only be computed for voxels damaged in more than 2
      people. A large number for this threshold can increase your statistical
      power, as you will only compute statistics for voxels that are commonly
      injured. However, larger values will fail to detect rarely damaged
      regions that are reliable predictors of deficit. Note that this value
      is based on the total incidence of lesions in a voxel, regardless of
      behavioral performance.

**Compute results**

Next, we we compute our statistical results. You can conduct
some statistics by choosing items in the Draw/Statistics menu of
MRIcron. However, here we describe using some of the new features in
NPM that are not yet available in MRIcron - specifically, NPM can
conduct permutation thresholding and the Brunner and Munzel test. To
conduct these statistics,
you need to download and install npm.exe - this only works on the
Windows operating system.

1. First go to the 'Options' menu and set the permutations to None.
   Permutation thresholding can be useful, but it will take at least 1000
   times longer than a normal False-Discovery Rate corrected threshold,
   and is typically less sensitive. Therfore, for you first glance at your
   data, turn this feature off.
2. For analyzing the continuous data:

1. Go to 'Option' menu and click 'Tests' - make sure the 'Brunner Munzel' is checked and the t-test and Welch test are unchecked.
   Our data is not normally distributed, so we will use a non-parametric
   test (using the permuted Brunner Munzel rank order statistic).
2. Click the File/BinaryImagesContinuousGroups command. Select the continuous.val file.

3. For analyzing the binomial data:

- Click the File/BinaryImagesBinaryGroups command. Select
  the binomial.val file. This will use the Liebermeister measure (a more
  sensitive binomial test than Chi-Squared or Fisher's Exact test, see Seneta and Phipps, 2001; Phipps, 2003).

4. NPM will now compute the requested tests. It will create
   overlap images of all your patients (e.g. sum.nii.gz) and a statistical
   map (BM.nii.gz for continuous data, L.nii.gz for binomial data).

**Viewing results**

We can open up the statistical maps generated and place them
on top of an anatomical scan. If your lesion maps were aligned to
stereotaxic MNI space, you can open them on top of one of the standard
templates (File/OpenTemplates/ch2). Here is a quick guide:

1. Launch MRIcron and choose File/OpenTemplates/ch2bet as our background image
2. Choose Overlay/Add and choose the statistical map created in the previous step (e.g. C:\dataset\binL.nii.gz).
3. When MRIcron detects a statistical map, it calculates the
   p-values
   for each test in order to determine the false discover rate (FDR)
   threshold - e.g. how much robust signal is present in your data.
   MRIcron displays a histogram of the Z-scores. Note in the example
   below, most of the data has positive Z scores suggesting a robust
   signal (if our data was merely noise, we should see a bell-shaped
   distribution with a mean of 0, instrate the mean Z score is around 3).
4. Next, MRIcron displays the overlay. Note at the bottom of the
   screen the software reports the critical values: the p05/p01 values
   correspond to uncorrected p<0.05 and p<0.01 values that are very
   liberal (these tests will make many false alarms, e.g. here we have
   conducted 16082 tests, so p05 should result in many false positives).
   The fwe05 and fwe01 values correspond to the Bonferroni-corrected
   values: this test is very conservative, and you will often fail to
   detect real effects. The FDR05 and FDR01 results reflect the False
   Discover Rate - e.g. a FDR05 should show around 20 real activations for
   every false positive. Note that when there is very little or no signal,
   FDR is as conservative as Bonferroni, but it is adaptive to the actual
   signal in your dataset. Note that you can select the image thresholding
   and cutoff for your overlay, as described in the  MRIcron manual. Note that by default,
   my software loads statistical maps with thresholds from FDR05 to FDR01,
   unless there is insufficient signal, in which case it uses the
   uncorrected 0.05...0.01 values. Also note that the current overlay is
   set to appear in a monochromatic red color scheme.
5. We can repeat steps 2..4 to load multiple overlays, to compare
   different statistical tests. For example, clicking on the
   'tutorialfmri.bat' icon will launch MRIcron and load to overlapping
   regions of interest. By using the Overlay/TransparencyOnOtherOverlays
   command we can view both of these overlays simultaneously.
